# Supplementary material for: Oro-faecal transmission of SARS-CoV-2: A systematic review of studies employing viral culture from gastrointestinal and other potential oro-faecal sources and evidence for transmission to humans
Source: Epidemiol Infect. 2024 Nov 12;152:e138. doi: 10.1017/S0950268824001481 (PMC11574600; doi:10.1017/S0950268824001481)
Supplement: Gandini et al. supplementary material 3 — Gandini et al. supplementary material [file S0950268824001481sup003.docx]

Table S1. Main findings of the included studies.

| Study | Ct values (or log copies, if Ct not reported) for faecal samples | Selection of samples subjected to viral culture attempt | Method used to identify viruses growing in cell culture, if any observed | Growth on culture (positivity rate) | Data on respiratory samples from the relevant study participants*: sample collection schedule, Ct values of RT-PCR. | | Data on symptoms on the relevant study participants: timing of symptom assessment, symptoms reported, signs observed |
| --- | --- | --- | --- | --- | --- | --- | --- |
| Akiyama, 2022 | patient 1:  day 18 +ve PCR stool, viral load 3.51 log10 copies/g; day 22 +ve PCR stool, viral load 7.83 log10 copies/g, day 24 +ve PCR stool, viral load 8.28 log10 copies/g, day 26 -ve PCR stool; day 30 -ve PCR stool.  patient 2:  day 16 +ve PCR stool, viral load 4.16 log10 copies/g, day 34 -ve PCR stool, day 38 -ve PCR stool.  patient 3: day 20 +ve PCR stool, viral load 5.36 log10 copies/g, day 30 +ve PCR stool, viral load 3.20 log10 copies/g.  patient 4: day 2 +ve PCR stool, 5.17 log10 copies/g, day 6 +ve PCR stool, viral load 4.39 log10 copies/g. | NR | NR | 0/8; Cell culture in Vero E6 /TMPRESS2 cells of 8 PCR +ve stool samples was performed over 4 weeks and no SARS-CoV-2 replication was observed. | Patient 1: day 16, PCR +ve, 5.14 log10 copies/ml, day 18 +ve 3.19 log10 copies/ml, day 22 +ve 3.86 log10 copies  patient 2: day 6 +ve 5.56 log10 copies, day 8 +ve 5.02 log10 copies, day 18 +ve 2.76 log10 copies, day 20 -ve.  patient 3:  day 6 +ve 4.95 log10 copies, day 8 +ve 5.29 log10 copies, day 16 +ve 4.01 log10 copies, day 18 +ve 2.69 log10 copies, day 20 +ve 3.08 log10 copies, day 34 -ve.  patient 4: day 2 +ve 7.53 log10 copies, day 6 +ve 2.20 log10 copies, day 10 +ve 4.20 log10 copies, day 12 -ve, day 14 +ve 2.12 log10 copies. | | Patient 1: 83 year old male, severe disease. Diarrhoea. patient 2: 63 year old male, critical disease. Diarrhoea. patient 3: 74 year old male, critical disease. Diarrhoea. patient 4: 53 year old male, moderate disease. No diarrhoea. |
| Albert 2021 | **Pt1 day1: N1=ND, N2=ND; day2: N1=36.8, N2=ND; day3: N1=34.2, N2=37.3. Pt2 day1: N1=37.0, N2=41.3. Pt3 day1: N1=34.1, N2=35.9, day4: N1=ND, N2=ND. Pt4 day1: N1=32.5, N2=35.1, day4 N1=ND, N2=ND. Pt5 no faecal samples. Pt6 day1: N1=37.9, N2=39.9, day3 N1=39.0, N2=39.4, day7: N1=ND, N2=40.6. Pt7 day1: N1=38.6, N2=ND. Pt8 day1 N1=ND, N2=42.7. | NR. "faecal...samples with highest RNA... were used to inoculate Vero E6 cells". | RNA in cell culture supernatant subjected to RT-qPCR, looking for potential increase over time. | None detected. "We were unable to detect evidence of replication in our assays." No. of faecal samples from 5 patients subjected to viral culture is unclear; appeared to be 8 including only undiluted specimens. | NR | | Timing of symptoms assessment not reported. Pt1 headache, odynophagia, Pt2 fever, headache, Pt3 fever, headache, Pt4 fever, headache, respiratory symptoms, Pt5 fever, headache, respiratory symptoms, odynophagia, GI symptoms, Pt6 fever, headache, respiratory symptoms, GI symptoms, Pt7 fever, headache, respiratory symptoms, Pt8 headache, odynophagia, respiratory symptoms, GI symptoms. |
| Cerrada-Romero 2022 | median 31.2 (range 24.5 to 39.6) in first week; median 34.5 (27.2 to 39.1) in second week. | 31 RNA positive stool samples. 62 patients, 79 stool samples. 27/62 patients had RNA positive stool samples. | RT-PCR on the cell supernatant | 0/31 | No differences in viral load in NP swabs between patients with stool samples testing positive (7.10 Log10 copies/mL [IQR 4.46 to 9.07]) or negative (7.81 Log10 copies/mL [IQR 6.13 to 9.01]); stool samples collected during 1st 2 weeks. Ct values in contemporaneous NP swabs ranged from 17 to 30 (median 21.7) and from 11.3 to 37.8 (median 33.3), respectively; Ct values were lower in NP swabs than in stool samples in the 7 pts tested in week 1, and in 6/9 pts in wk 2 post-onset. However, NP swabs were negative in 3/9 and 3/6 pts tested in weeks 3 to 4 and 2 to 4 months after symptom onset. | | Pneumonia was detected on admission in all pts whose stool samples tested negative, and in 75% of those pts whose stool samples tested positive. Final Covid-19 WHO score moderate severity: 15 (93.8%) 30 (96.8%) in patients with SARS-CoV-2 RNA in stool samples compared to those without it. |
| Dergham 2021 | NR overall. For 2 samples yielding successful cell culture: Ct = 17.29, Ct = 16.22 | 106 samples (from 46 patients) were available for cell culture. | qPCR and WGS (methods reported). Once CPE was detected in a well, the supernatant was collected. 20 microliters were used for qPCR for confirmation; then whole genome sequencing. | 2/106 | For all 46 patients: NR For the immunocompromised patient: COVID-19 pneumonia was diagnosed on chest CT. NP SARS-CoV-2 PCR was positive only once on 15 April (Ct of 33.5, 2.099 copies/mL) and was negative on 21, 22 and 28 April. | | For all 46 patients: NR For the immuoncompromised patient: in previous 10 days asthenia, loss of appetite, diarrhoea, and weight loss without respiratory symptoms. Admission revealed acute kidney injury and mild inflammation. C reactive protein normalized on 18 April. |
| Fumian 2021 | N1 primer: mean 34.1 (range 29.8 to 37.0); N2 primer: 5 not detected; among 5 positive, mean 35.5 (range 34.3 to 37.8) | Four RNA-positive samples from 4 patients with low Ct values (from 29.8 to 32.4) were submitted to virus isolation in Vero E6 cell cultures. | CPE was evaluated using a microscope. Additionally, cell supernatant from the three passages was obtained, the RNA extracted and tested by PCR for the presence of SARS-CoV-2. | 0/4 | No information reported on respiratory clinical signs, or molecular diagnostic of SARS‐CoV‐2 in other clinical specimens. | | All included patients had acute gastroenteritis. No data on respiratory symptoms was available (the cohort was defined by gastrointestinal symptoms) |
| Jeong 2020 | Pt 2: 1.17 +/- 0.32 log copies, Pt 4: 2.18 +/- 0.11 log copies, Pt 5: 2.01 +/- 0.28 log copies. | Not reported. | Cells were monitored daily for CPE. RT-PCR performed on cell supernatants of infected cultures. | 0/3 | Pt 2: NP 1.18 ± 0.12 log copies, Pt 4 NP 1.15 ± 0.03 log copies, Pt 5 1.43 ± 0.42 log copies | | 1 pt mild disease; 3 pts severe disease with pneumonia requiring oxygen supply; 1 pt critical with respiratory failure and shock Samples taken: Pt 1 day 8, recovery phase; Pt 2 day 13, recovery, Pt 3 day 11, recovery, Pt 4 day 15, acute illness, Pt 5 day 30, recovery. |
| Joshi, 2022 | 19.5–25.9 and 22.6–28.1 for E gene and ORF region of SARS-CoV-2 respectively | Specimens collected between 0 and 6 post onset day from 10 patients. | The viral RNA load in the faecal specimens was estimated using N gene based digital RT-PCR assay as per manufacturer’s instructions (SARS-CoV-2 N1 + N2 Primer Probe Assay Kit, Qiagen, Germany). Thermal cycling was performed at 50◦C for 40min for reverse transcription, followed by 95◦C for 2min and then 40 cycles of 95◦C for 05 s, and 60◦C for 30 s. Analysis of the viral RNA load in faecal specimens indicated 5526.2–15.2 copies per/μL of RNA. Faecal specimens of COVID-19 patients were subjected to RT-PCR, digital RT-PCR, Next Generation Sequencing (NGS), isolation in Vero CCL-81 cell lines according to an established protocol. Ct values of the faecal specimens ranged from 19.5–25.9 and 22.6–28.1 for E gene and ORF region of SARS-CoV-2 respectively. | 0/10 | All throat swab specimens were positive with Ct values between 18–27 and 21–29 for E gene and ORF region respectively | | Among ten patients, six were experiencing symptoms like fever, cough, sore throat, chest pain, wheezing, abdominal pain, vomiting/nausea, diarrhoea, loss of taste and smell, while the remaining four were asymptomatic |
| Lavania 2022 | Ct values in the faecal specimens were lower than in the throat specimens. | 55 faecal samples with low Ct; methods refer to a reference for the protocol and cultures only done if the Ct for the E gene was < 30; unclear if from 55 patients but assumed to be. | Reported in Sarkale et al 2020. Daily visual inspection for CPE. Cell supernatant of passage 1 was taken on day 3 of culture and tested by RT-PCR using E and RdRp gene; also of passage 2 at day 4. | 0/55 | NR | | Pts were admitted with myalgia, sore throat, fever, and cough hypoxia. 15.4% pts showed GI symptoms such as diarrhoea and abdominal pain. 62 (67.39%) pts, who tested positive for SARS-CoV2 RNA in feces, were identified as asymptomatic cases. 108 (38.5%) pts were mild cases: 60/108 of whom tested positive for viral RNA in feces; 48/108 tested negative. 80 pts were severe cases with hypoxia, SPO2 <94%. 51/80 tested positive for SARSCoV-2 viral RNA in feces; 29/80 tested negative. No significant association between GI symptoms, severity, and faecal viral load. The median (interquartile range [IQR]) duration time between symptom onset and 1st positive RT-PCR faecal test result was 11 (7 to 13) days. The median time of intervals from disease onset to sampling was 9 days (range, 4–10 days. |
| Nogueira, 2022 | From 22.6 to 37.4 | Aliquots of positive samples from 7 patients were kept at 4 C (which preserves virus infectivitybetter than dissolving in 0.9% NaCl and/or freezing at -80^0^ C) for further testing of viral viability in cell culture. | The Vero-E6 (ATCC CRL-1586) cell line, which is permissive for the propagation of SARS-CoV-2, was used in the present study. Cells were grown in 1 DMEM (Dulbecco’s Modified Eagle’s Medium, Gibco, Billings, MT, USA) + GlutaMax (Gibco, Billings, MT, USA) + 10% fetal bovine serum (FBS, Gibco, Billings, MT, USA) + 1 Penicillin/Streptomycin (P/S; Gibco, Billings, MT, USA) + 1 sodium pyruvate (SP) (Gibco, Billings, MT, USA) + 1 non-essential amino acids (NEAA; Sigma Aldrich, St. Louis, MO, USA), and were cultured at 37 C in a 5% CO2 incubator. In two technical replicates, 500 L of DMEM 0 and 300 L of the stool suspension, were pipetted into each well and incubated for 1 h at 37 C. The medium was then removed, the cells were washed with DMEM + 2% FBS + P/S + SP + NEAA and incubated in 2 mL of the same medium at 37 C in a 5% CO2 incubator for a total of 7 days. The cells were inspected for potential cell lysis by light microscopy on a daily basis. At 0, 4, and 7 dpi, 140 L aliquots of the medium were taken from each well and frozen at 􀀀80 C for further RT-qPCR analysis. The SARS-CoV-2 BU2-NS P3 strain (a kind gift of Dr. Jan Weber, IOCB, Prague, Czechia) was used as a positive control, while non-infected Vero-E6 cells (mock) served as a negative control. Controls were processed in the same way as described above. | 0/20 | NR | | Included mainly elective surgery, gastrointestinal symptoms, and various other problems mostly unrelated to acute infection. |
| Pedersen, 2022 | 14/33 rectal swabs positive. Median concentration of SARS-CoV-2 RNA was 13,014 IU/ml (IQR 6824 to 34,403) from 28 patients. | All collected. | Supernatants analysed by RT-PCR on inoculation and on days 3 and 6 to detect viral proliferation and confirm viral identity. | 0/33 in rectal swabs contained culturable virus. In the corresponding respiratory samples, 11/33 samples contained culturable virus. For 3 of the culturable respiratory samples, the paired rectal sample was negative by RT-PCR) | 30/33 pharyngeal swabs positive. Median concentration of SARS-CoV-2 RNA was 2,190,186 IU/ml (IQR 237,929 to 12,446,910) | |  |
| Ribeiro, 2022 | Ct-values of positive-RS samples ranged from 25.2 to 36.4, with a median value of 33.5. | 333 rectal swabs samples, with a median of two samples (ranging from 1 to 5) per individual, collected during home visits using a sterile cotton swab and immersed in sterile tubes containing viral transport media (VTM). | sgN mRNA are detected from RS samples, which provides evidence of active SARS-CoV-2 replication in intestinal cells. As sg mRNA is transcribed only in infected cells and is not packaged into virions, its detection indicates the presence of actively infected cells in samples. evidence of replication and sg mRNA detection demonstrated by increasing viral load after viral isolation in cell culture of a clinical RS sample (S4), passage 1 (S3), passage 2 (S2), and positive control (S1). The rectal swab sample (id_30771) was collected from a 68-year-old woman who tested positive for SARS CoV-2, with a Ct value of 25.2. A cytopathic effect (CPE) was observed after 3 days of incubation, and the Ct value of the infected-culture supernatant was 9.1. A second-round passage (2P), performed three days post-infection, generated a viral stock | 1/ 14 (presence of infectious SARS-CoV-2 from 1 out of 14 rectal swab samples inoculated in Vero cells. Evidence of replication and sg mRNA detection demonstrated by increasing viral load after viral isolation in cell culture of a clinical RS sample (S4), passage 1 (S3), passage 2 (S2), and positive control (S1)) | 50.2% (350/697) nasopharyngeal swabs positive | | Not provided |
| Wölfel 2020 | NR | NR | Culture supernatant was collected after 0,1,3,5 days and used in RT-PCR analysis. | 0/13 | NR for the 4 patients providing the 13 faecal samples. However, overall: "The average virus RNA load was 6.76 × 105 copies per whole swab until day 5, and  the maximum load was 7.11 × 108 copies per swab. Swab samples taken  after day 5 had an average viral load of 3.44 × 105 copies per swab". | | All had mild disease. 1/13 had intermittent diarrhoea. |
| Yao, 2020 | <=28 for all 3 faecal samples tested by viral culture from 3 patients from a cohort of 11 patients | Samples with Ct =<28 selected for cell culture. | Culture supernatant was tested by qRT-PCR analysis. | 3/3 | NR | | Severe disease in all 3 patients (they later recovered). |
| *those from whom faecal samples were obtained and subjected to viral culture. | | | | | |  |  |
| **sampling date reported as calendar date, unclear if this equals admission date or other factor. | | | | | |  |  |
| NR = not reported | | | | | |  |  |
