# Supplementary material for: Oro-faecal transmission of SARS-CoV-2: A systematic review of studies employing viral culture from gastrointestinal and other potential oro-faecal sources and evidence for transmission to humans
Source: Epidemiol Infect. 2024 Nov 12;152:e138. doi: 10.1017/S0950268824001481 (PMC11574600; doi:10.1017/S0950268824001481)
Supplement: Gandini et al. supplementary material 4 — Gandini et al. supplementary material [file S0950268824001481sup004.docx]

Table S2. Risk of bias assessment results.

|  | Were the criteria for diagnosing a case clearly reported and appropriate? | Was the reporting of patient/ population characteristics adequate? | Were the methods used to obtain RT-PCR results replicable and appropriate? | Was the study period, including follow-up, sufficient to investigate orofecal transmission? | Were the methods used to obtain viral culture results replicable and appropriate? | Were the analysis and reporting of the results appropriate |
| --- | --- | --- | --- | --- | --- | --- |
| Akiyama, 2022 | yes | yes | yes | N/A | unclear | yes |
| Albert 2021 | yes | yes | unclear | N/A | unclear | yes |
| Cerrada-Romero C 2022 | yes | yes | yes | yes | unclear | yes |
| Dergham J 2021 | yes | yes | yes | yes | unclear | yes |
| Fumian TM 2021 | yes | yes | yes | yes | unclear | yes |
| Jeong 2020 | yes | yes | unclear | N/A | unclear | unclear |
| Joshi 2022 | yes | yes | yes | N/A | yes | yes |
| Lavania M 2022 | yes | yes | yes | yes | unclear | yes |
| Nogueira 2022 | yes | yes | yes | N/A | yes | yes |
| Pedersen 2022 | yes | yes | yes | N/A | yes | yes |
| Ribeiro 2022 | yes | yes | No | N/A | yes | yes |
| Wang W 2020 | yes | unclear | yes | N/A | unclear | yes |
| Wölfel 2020 | yes | no | unclear | N/A | unclear | yes |
| Yao 2020 | yes | yes | yes | N/A | yes | unclear |
